# Acute liver failure associated with diffuse liver infiltration by metastatic breast carcinoma: A case report

**DOI:** 10.3892/ol.2013.1165

**Published:** 2013-01-30

**Authors:** SUCHANAN HANAMORNROONGRUANG, NAPAKORN SANGCHAY

**Affiliations:** Department of Pathology, Faculty of Medicine, Siriraj Hospital, Mahidol University, Bangkok 10700, Thailand

**Keywords:** diffuse parenchymal metastasis, acute liver failure, hepatic infiltration, liver metastasis, metastatic breast cancer

## Abstract

Diffuse parenchymal metastasis is an unusual pattern of liver metastasis that is capable of causing liver failure. In the present study, the authors describe malignant infiltration of the liver by primary breast carcinoma in an autopsy of a 49-year-old female who had a rapid onset of liver failure and died after three weeks. Ultrasonography and computed tomography (CT) scans of the abdomen, as well as macroscopic examination, failed to detect liver metastasis; while microscopic examination revealed diffuse tumor cells with a loss of E-cadherin expression infiltrating into the liver parenchyma. The prognosis of liver failure associated with malignant infiltration is extremely poor; the survival time of patients is extremely low. Liver biopsy may be the most efficient technique for confirming the diagnosis during the patient’s life.

## Introduction

Although the liver is a common target for metastasis, acute or fulminant liver failure due to metastasis is a rare occurrence (1–7). Diffuse parenchymal metastasis is a rare pattern of liver metastasis that is correlated with hepatic failure and an extremely poor prognosis (1–8). Unlike the usual patterns of liver metastasis, radiological studies are typically not able to detect this type of pattern (1,3–5,7). Therefore, metastasis is frequently diagnosed by autopsy (3). In the present study, the authors describe a case of acute liver failure correlated with diffuse liver infiltration by metastatic carcinoma in a 49-year-old female with a history of breast cancer.

## Case report

### Clinical summary

A 49-year-old Thai female was admitted to hospital due to a high grade fever, jaundice and abdominal pain in the right upper quadrant. Four weeks prior to admission, the patient suffered from malaise, anorexia and weight loss. An initial investigation revealed that the patient had anemia (Hct, 23%) and abnormal liver function test results (total bilirubin, 75.24 *μ*mol/l; direct bilirubin, 34.2 *μ*mol/l; AST, 250 U/l; ALT, 63 U/l; ALP, 198 U/l). The patient received treatment for chronic cholecystitis and hepatitis; however, the symptoms progressed and the patient developed thrombocytopenia and coagulopathy. The liver function test results also worsened (peak values: total bilirubin, 890.91 *μ*mol/l; direct bilirubin, 598.5 *μ*mol/l; AST, 1152 U/l; ALT, 114 U/l; ALP, 845 U/l; GGT, 727 U/l), while both ultrasonography and computed tomography (CT) scans of the abdomen were not able to detect any specific hepatic lesions. No evidence of viral hepatitis or any identifiable source of infection was observed. The patient’s condition deteriorated further; hepatic encephalopathy developed and the patient died within the third week following the onset of acute hepatic failure. The patient had a history of breast carcinoma stage I and had been treated by modified radical mastectomy (MRM), and had remained disease-free for 10 years following treatment.

### Pathological findings

**Figure 1:**
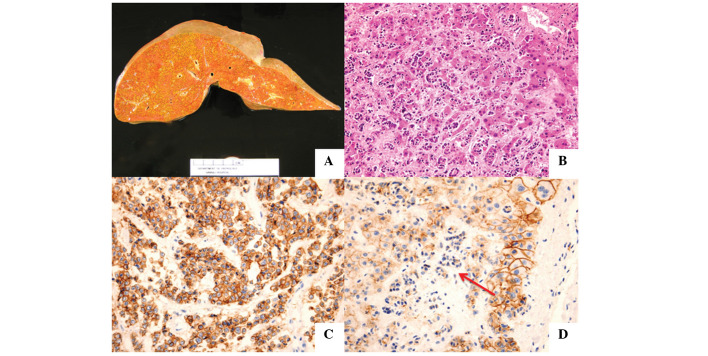
(A) Image of the unfixed liver revealed hepatomegaly without gross metastasis. (B) Microscopic examination of the liver revealed diffuse infiltration by pleomorphic cells with some glandular formation. (C) Immunohistochemical staining for E-cadherin expression in the metastatic nodule in the lung was strongly positive, (D) that of metastatic tumor cells in the liver was negative (arrow).

The gross appearance of the liver revealed that the patient had hepatomegaly (2,150 gm). The liver capsule was smooth, the cut surfaces of which showed non-homogeneous dark brown and yellow tissue and no mass-forming lesions (Fig. 1A). An ill-defined yellow-brown nodule (4 cm in diameter) was observed at the pancreas, and multiple small yellow-brown nodules were found at the pleural surface and parenchyma of both lungs. Other abnormal gross findings included splenomegaly and pulmonary congestion. A previous surgical scar from the MRM was detected without any abnormality in both breasts or axillary regions.

Microscopic examination revealed diffuse infiltration of the liver parenchyma by pleomorphic cells with some glandular formation (Fig. 1B). Extensive necrosis of hepatocytes was noted. The tumor cells were positive for CK7, ER and PR, while negative for CK 20 and TTF-1. These results support the diagnosis of primary breast carcinoma.

Metastatic tumor infiltration was also detected in the spleen and both adrenal glands. Sections from nodules in the pancreas and the lungs demonstrated metastatic carcinoma. In addition, microscopic foci of metastasis were observed in the uterine cervix, appendix, bone marrow, thyroid and intrathoracic lymph nodes. No evidence of carcinoma was identified in the breast tissue samples. Additional abnormal histological findings included pulmonary congestion with edema and focal hemorrhage, myocardial hypertrophy, acute tubular necrosis of both kidneys and hypoxic-ischemic changes in the brain tissue.

Immunohistochemical staining for E-cadherin was also performed. Notably, tumor cells in the lung nodule strongly expressed E-cadherin (Fig. 1C), while tumor cells in the liver did not (Fig. 1D).

## Discussion

With the exception of the lymph nodes, the liver is considered to be the most frequent site of metastasis (9–11). In breast cancer, the liver is also one of the major metastatic targets along with the lungs and bone (1,9,12). The most common pattern of liver metastasis is the formation of discrete multiple nodules followed by a single nodule, while diffuse tumor invasion into the liver parenchyma or diffuse parenchymal metastasis is less common (3,4,12). Hematological malignancies are recognized to be the most common cause of diffuse parenchymal metastasis (1,3,4). This metastatic pattern has also been identified in many primary neoplasms, including breast, lung, stomach, colon, pancreatic, nasopharynx, urothelial, uterine and malignant melanoma (3,4,7,8).

Acute liver failure rarely occurs in typical liver metastasis, although abnormal liver enzymes may be detected (3,4,7), whilst this condition is more common in diffuse parenchymal metastasis (1,7). This complication is speculated to be caused by extensive hepatocellular necrosis resulting from pressure atrophy and interference of the vascular supply (4,6–8,13). Radiological studies including ultrasonography, CT and MRI scans are usually not able to identify diffuse parenchymal metastasis (1,3–5,7), thus increasing the difficulty in the diagnosis. Radiological findings resembling cirrhosis have also been reported (1,7,14). To confirm the diagnosis, histological examination is necessary and a liver biopsy may be useful (3,5,7).

The prognosis of acute liver failure secondary to malignant infiltration is extremely poor; the majority of patients do not survive shortly after the onset of liver failure (1–4,7,8). According to a review by Allison *et al*, concerning 21 reported cases of acute liver failure due to metastatic breast carcinoma, 18 cases died within three days to two months (7). Non-specific prodromal symptoms, such as malaise, weight loss, right upper quadrant abdominal pain and fever, are always observed to be present at least two to four weeks prior to the onset of liver failure (3,4).

The underlying mechanism of diffuse parenchymal metastasis remains unknown. Allison *et al* proposed a loss of cell surface adhesional molecule expression; the three cases in their study described as diffuse intrasinusoidal hepatic metastasis did not express both E-cadherin and CD44, which are glycoproteins involved in cell-cell and cell-extracellular matrix adhesion (7). In the present case, a difference in E-cadherin expression in the two different areas of metastasis was observed; the tumor cells in the metastatic nodule in the lung stained positive for E-cadherin, while the infiltrating tumor cells in the liver stained negative. These findings support the hypothesis that a loss of cell surface adhesional molecule expression is involved in facilitating diffuse parenchymal metastais.

In conclusion, although malignant infiltration of the liver is rare, it ought to be considered as a differential diagnosis in patients with acute hepatic failure. Liver biopsy may be the most effective technique to confirm the diagnosis during the patient’s life.
